# Childhood traffic-related air pollution and adverse changes in subclinical atherosclerosis measures from childhood to adulthood

**DOI:** 10.1186/s12940-021-00726-x

**Published:** 2021-04-14

**Authors:** Shohreh F. Farzan, Rima Habre, Phoebe Danza, Frederick Lurmann, W. James Gauderman, Edward Avol, Theresa Bastain, Howard N. Hodis, Carrie Breton

**Affiliations:** 1 Department of Preventive Medicine, Keck School of Medicine of University of Southern California, 2001 N. Soto Street, Los Angeles, CA 90089 USA; 2 Sonoma Technology Inc., Petaluma, CA USA; 3 Department of Medicine, Keck School of Medicine of University of Southern California, Los Angeles, CA 90089 USA; 4 Atherosclerosis Research Unit, University of Southern California, Los Angeles, CA 90089 USA

**Keywords:** Carotid intima media thickness, Air pollution, Traffic, Atherosclerosis, Childhood

## Abstract

**Background:**

Chronic exposure to air pollutants is associated with increased risk of cardiovascular disease (CVD) among adults. However, little is known about how air pollution may affect the development of subclinical atherosclerosis in younger populations. Carotid artery intima-media thickness (CIMT) is a measure of subclinical atherosclerosis that provides insight into early CVD pathogenesis.

**Methods:**

In a pilot study of 70 participants from the Southern California Children’s Health Study, we investigated CIMT progression from childhood to adulthood. Using carotid artery ultrasound images obtained at age 10 and follow-up images at age 21–22, we examined associations between childhood ambient and traffic-related air pollutants with changes in CIMT over time and attained adult CIMT using linear mixed-effects models adjusted for potential confounders.

Average residential childhood exposures (i.e., birth to time of measurement at 10–11 years) were assigned for regional, ambient pollutants (ozone, nitrogen dioxide, particulate matter, interpolated from regulatory air monitoring data) and traffic-related nitrogen oxides (NO_x_) by road class (modeled using the CALINE4 line source dispersion model). Traffic density was calculated within a 300-m residential buffer.

**Results:**

For each 1 standard deviation (SD) increase in childhood traffic-related total NO_x_ exposure, we observed greater yearly rate of change in CIMT from childhood to adulthood (β: 2.17 μm/yr, 95% CI: 0.78–3.56). Increases in annual rate of CIMT change from childhood to adulthood also were observed with freeway NO_x_ exposure (β: 2.24 μm/yr, 95% CI: 0.84–3.63) and traffic density (β: 2.11 μm/yr, 95% CI: 0.79–3.43). Traffic exposures were also related to increases in attained CIMT in early adulthood. No associations of CIMT change or attained level were observed with ambient pollutants.

**Conclusions:**

Overall, we observed adverse changes in CIMT over time in relation to childhood traffic-related NO_x_ exposure and traffic density in our study population. While these results must be cautiously interpreted given the limited sample size, the observed associations of traffic measures with CIMT suggest a need for future studies to more fully explore this relationship.

**Supplementary Information:**

The online version contains supplementary material available at 10.1186/s12940-021-00726-x.

## Background

Cardiovascular disease (CVD) remains a growing public health issue across the globe, and CVD incidence is predicted to increase steadily over the coming decades [1, 2]. Despite more effective treatments and greater awareness of risk factors, CVD continues to be the leading cause of death worldwide, accounting for nearly a third (~ 18 million) of all deaths each year [3]. More than 80% of CVD has been attributed to modifiable factors, including environmental exposures [4–10]. Given the high incidence of CVD, even small increases in risk due to environmental factors may translate to a considerable increase in the number of individuals who develop CVD.

Air pollution has emerged as a key contributor to many chronic diseases, including CVD, and a large body of evidence supports both the acute and chronic cardiovascular effects of air pollution exposure [8, 9, 11–19]. Epidemiological and experimental studies have linked air pollutant exposures to multiple risk factors for CVD [20–27], including carotid artery intima media thickness (CIMT), a widely used measure of subclinical atherosclerosis [28, 29], and carotid arterial stiffness (CAS), a measure of endothelial function indicative of vascular health [30]. While the majority of studies have focused on the role of ambient pollutants, proximity to traffic also has been associated with atherosclerosis, as measured by CIMT and/or coronary artery calcification, in several studies among adults [31–34]. Further, two studies of long-term exposure to fine particulate matter (PM_2.5_) and traffic-related pollutants found associations with accelerated progression of atherosclerosis, as measured by increases in CIMT over time [32, 35]. Together, these studies provide a strong body of evidence supporting a role for ambient and traffic-related air pollutants in the development of CVD by promoting atherosclerosis, the key underlying pathological process that leads to clinical cardiovascular events.

Despite the relatively strong evidence linking air pollution and CVD in adults, very little is known about the origins of CVD in early stages during childhood and adolescence, particularly in relation to air pollution. A limited number of studies suggest that air pollution exposure may be related to adverse cardiovascular changes in children and young adults [21, 36–40]. A number of studies have observed that prenatal and early air pollution exposures are related to adverse changes in childhood cardiovascular risk factors, such as blood pressure (BP) and body mass index (BMI) [41–46]. In studies of college students, prenatal exposure to fine particulate matter (PM_2.5_) has been associated with increased CAS, while childhood exposure to ozone has been associated with greater CIMT in early adulthood [21, 22]. While changes in childhood cardiovascular risk factors have been associated with increases in CIMT in later life [47–50], to our knowledge, no studies have examined changes in subclinical atherosclerosis over time in younger populations, particularly at the transition from childhood to adulthood, nor have others examined the potential role of air pollutants in early atherosclerosis progression during this critical life stage.

The Southern California Children’s Health Study (CHS) is one of the largest and most comprehensive investigations of the long-term effects of air pollution on children’s health [51–54]. In a study of 70 CHS participants, we measured CIMT in both childhood and adulthood and examined longitudinal changes in this measure of subclinical atherosclerosis over this critical development time. Leveraging the extensive and well-characterized exposure data available in the CHS, we assessed the impact of both ambient and traffic-related air pollutants on patterns of CIMT change over time and on attained CIMT level in adulthood.

## Methods

### The Southern California Children’s Health Study

The Children’s Health Study (CHS) began in 1993 and since its initiation, five discrete CHS cohorts have been established to evaluate the health effects of air pollution in school children who were followed prospectively from 6 until 18 years of age. The CHS is one of the largest and most comprehensive investigations of the long-term effects of air pollution on the respiratory health of children, and portions of the CHS have been followed for over 20 years [51, 52, 55, 56]. As part of the NIH Environmental Influences on Child Health Outcomes (ECHO) consortium (UH3OD023287), we are actively following participants from the fifth CHS cohort (“Cohort E”), in which approximately five thousand children from kindergarten and first grade in 2002–2003 were enrolled. These children were recruited from 13 communities, representing a range of levels and mixtures of regional ambient air pollutants, including particulate matter (PM), nitrogen dioxide (NO_2_) and ozone (O_3_) [55, 56]. Existing childhood data (from age 5 through 18) collected on CHS participants include parental sociodemographic factors, annual health questionnaires, annual measurements of height and weight to obtain validated measures of BMI, as well as multiple years of pulmonary function testing and exhaled nitric oxide measurements. Of those enrolled in CHS Cohort E, a subset children from 8 CHS communities were selected for a carotid artery ultrasound assessment during their annual visit at approximately 10–11 years of age (school year of 2007–2008) [36]. Children were selected to receive a CIMT measurement in childhood based on parental report that the child lived in a non-smoking household and had lived in the same residence from birth until time of CIMT measurement in one of 8 selected CHS communities. Children meeting these criteria in were invited to participate and were measured upon receipt of parental consent, resulting in 737 children with CIMT measurement. For the purpose of this study, we contacted only participants from cohort E who had a childhood carotid artery ultrasound examination to participate in this follow-up study [36]. All participants provided written informed consent prior to enrollment. All protocols and study materials were approved by the University of Southern California’s Institutional Review Board.

### In-person assessment of CHS participants in early adulthood

Participants were re-contacted by phone and/or email approximately 11 years after their initial carotid ultrasound and invited to participate in an in-person follow-up visit at our ECHO study clinic. At the time of this study, approximately 530 Cohort E CHS participants of the original 737 children with CIMT measurements had recently updated contact information (e.g. phone number and/or email updated within 5 years), and records indicated that they lived in CA (for exposure assessment purposes, i.e. CALINE4 traffic estimates) within driving distance (~ 2.5 h) of our study clinic. From those 530 individuals, a convenience sample of the first 70 participants who to agree to participate in an in-person assessment were enrolled and provided written informed consent prior to assessment. While participants were not randomly selected for this pilot study, key characteristics of these 70 individuals, including community of origin, race/ethnicity and sex, are similar to the distributions observed in the original subset of 737 individuals with carotid ultrasound in childhood. Participants were asked to complete a number of questionnaires developed as part of the ECHO program that included questions about demographics, current lifestyle and habits, such as personal cigarette smoking and family health history including cardiovascular disease such as hyperlipidemia, hypertension, stroke and coronary artery disease. Trained study coordinators conducted duplicate measurements of weight and standing height, using standard protocols [57]. A third measurement of weight and/or height was taken if the first two measurements differed by more than 0.05 kg or 1 cm, respectively. Height and weight measures were used to calculate body mass index (BMI; kg/m^2^), which in turn was used to categorize study subjects (underweight/normal< 25 kg/m^2^, overweight (≥25 to 29.99 kg/m^2^), obese, ≥30 kg/m^2^), according to CDC guidelines [58]. After 5 min of rest, systolic and diastolic blood pressure were measured in triplicate with 1-min rest intervals between measurements using a GE Carescape Dinamap V100. Blood pressure measurements were averaged for analyses.

### Carotid artery ultrasound imaging and measures of atherosclerosis progression

Carotid artery ultrasound images were obtained from young adult participants during the in-person clinic visit (mean age at assessment = 21.9 (SD 0.4) years). Imaging was conducted at the USC Atherosclerosis Research Unit (ARU) by the same technician who conducted all childhood ultrasound scans at age 10–11. Acquisition of ultrasound images and subclinical carotid artery atherosclerosis was conducted with standardized procedures and technology specifically developed for longitudinal measurements of atherosclerosis at the ARU [59–62]. In brief, high resolution B-mode ultrasound carotid artery images were acquired using a Siemens Acuson CV70 (Mountain View, CA) ultrasound imaging system using a linear array 7.5 MHz transducer [61]. Single lead electrocardiogram (ECG) and ultrasound images were simultaneously recorded to ensure that vascular measurements occurred at the same time in cardiac cycle. The right carotid artery was imaged where the jugular vein and carotid artery were imaged transversely and then longitudinally with the former vessel stacked above the latter providing internal anatomical landmarks used for reproducing ultrasound transducer angulation and replicate image acquisition. Simultaneously visualizing the previously acquired ultrasound image from which CIMT was measured during childhood (10–11 years of age), internal anatomical landmarks were used to acquire the replicate ultrasound image for measurement of adult CIMT (20–21 years of age). Ultrasound image processors were blinded to all exposure information to avoid bias.

Arterial dimensions including far wall CIMT were measured at sub-pixel resolution using automated computerized edge detection software (Patents 2005, 2006, 2011) [59, 60]. CIMT was determined as the average of 70 to 100 measurements between the intima-lumen and media-adventitia interfaces along a 1-cm length just proximal to the carotid artery bulb at the same point of the cardiac cycle. This method standardizes the location and the distance over which CIMT is measured, ensuring comparability within and across participants [59, 60]. CIMT is a widely used measure of subclinical atherosclerosis that has been associated with risk of CVD events [28, 29]. Our CIMT methodology has been correlated with change in coronary artery disease assessed by quantitative coronary angiography and is predictive of clinical coronary events [28, 63].

### Calibration of ultrasound imagers and quality assurance

Childhood and adult ultrasound images were obtained using different ultrasound imaging equipment. Childhood ultrasound images at age 10–11 were obtained on a GE LOGIQ imager [36], which has limitations for long-term future use (i.e., outdated equipment with limited image storage capacity), while adult ultrasound images were obtained using current imaging technology (Siemens Acuson CV70) by the same ultrasound technician. To ensure comparability between childhood and adult images, duplicate ultrasound images were obtained from 29 adult participants using both the GE and Siemens ultrasound imaging systems. Reliability of outcome measurements performed across the ultrasound imaging systems was explored by calculating intra-class correlations between all measures that were performed in duplicate on both the GE and Siemens systems. Reliability of measurements between image analysts was also calculated for a subset of images.

### Exposure assessment

Residential histories were compiled and geocoded at the monthly level for all participants, starting from pre-conception through the date of the adult follow-up assessment. Timelines integrate several information sources (e.g. birth certificates, questionnaires, etc.) and explicitly quantify spatial and temporal uncertainty in location ascertainment. These timelines form the basis of all air pollution exposure assessments. For the purposes of this study, we focused on childhood exposures spanning the period from birth until the time of first ultrasound assessment in childhood (~age 10–11 years). Ambient exposures to regional PM_10_, PM_2.5_, O_3_ and NO_2_ were assigned at a monthly level (averaged from daily with 75% completeness criterion) using inverse-distance squared (IDW2) spatial interpolation based on the US Environmental Protection Agency’s regulatory air monitoring network data and CHS measurements. Given the relatively high monitoring network density in southern California compared to other regions in the United States [64], this approach provides adequate prediction of regional, background exposures and mainly captures their temporal variability.

Traffic density within a 300-m buffer around the residence was calculated using annual average daily traffic (AADT) volumes. Annual changes were scaled to the 2002 Southern California air basin fleet NOx emissions. The roadway network data was retrieved for all road classes from the ESRI Streetmap Premium national database. Traffic density was calculated across 4 road classes defined by Feature Class Codes (FCC) (freeway (FCC1), highway (FCC2), major collector (FCC3), minor collector and arterials (FCC4)).

We used the California Line Source (CALINE4) dispersion model [65] to estimate residential monthly NO_x_ concentrations from nearby on-road vehicular traffic on freeways (defined as FCC1 + FCC2 roads), non-freeways (FCC3 + FCC4 roads) and their total (as the sum of the two categories). This model is well suited for estimating vehicle emissions concentrations downwind of roadways [66, 67] and near-road freeway concentrations [68–71]. CALINE NO_x_ estimates by road class capture mainly spatial variability in emissions, traffic volume and roadway geometries as well as temporal variability in meteorology impacting dispersion of on-road vehicle emissions downwind.

### Statistical models

We calculated descriptive statistics for all variables of interest, including demographic characteristics, exposure metrics and outcome measures. Differences in CIMT change between demographic variable categories were assessed by t-test or one-way ANOVA. Trends in CIMT over time were visualized using scatter plots to compare outcome measurements at age 10–11 to measurements at age 21–22. Spearman correlations between all ambient and traffic-related pollutants were calculated.

Using linear mixed effects models adjusted for covariates, we examined each of the ambient and traffic-related air pollutant exposure variables with *attained* CIMT at the time of the adult ultrasound assessment, and the *yearly change* in CIMT from childhood to adulthood. Average childhood air pollutant exposures from birth to time of childhood CIMT measurement were determined by averaging monthly residential estimates of ambient and traffic-related pollutants. Yearly average change CIMT was determined by subtracting the child CIMT measurement value from the adult CIMT measurement value and dividing by total years between the two CIMT measurements. Effect estimates were scaled to a 1 standard deviation increase in the indicated exposure variable. Original study community (community at time of CHS enrollment in childhood) was determined to be a crucial study design variable, and both parental education (a proxy for socioeconomic status) and adult personal smoking behavior (categorized as never smokers, former smokers (no smoking within past 12 months), and recent smokers (any smoking within past 12 months)) were identified as important precision variables based on previous literature. Other potential confounders were then assessed by a one-by-one variable approach, and those that resulted in a pollutant-effect change larger than 10% were selected for inclusion in final models. BMI measures (i.e., adult BMI, child BMI and change in BMI over time) were each important (i.e. resulted in a pollutant-effect change larger than 10%) for both attained and yearly change in CIMT over time, but only adult BMI was included in final models, given the high degree of correlation between adult and child BMI (r = 0.71, *p* < 0.0001), as well as between adult and change in BMI over time (r = 0.75, *p* < 0.0001). Final multivariate linear mixed models included personal smoking behavior, parental educational attainment and adult BMI, with community of enrollment included as a grouping variable. Family history of CVD and blood pressure (e.g. adult systolic blood pressure or change in systolic blood pressure over time) were considered as possible confounders, but neither were associated with CIMT in our study population, and therefore not included in final models. However, a sensitivity analysis including both family history of CVD and blood pressure as covariates was performed. Exploratory analyses stratified by sex were also performed. Statistical significance was determined at *p* < 0.05 (two-sided). All analyses were performed using SAS 9.4 (SAS Institute, Inc., Cary, NC, USA).

## Results

In our study population of 70 CHS pilot participants, more than half of participants were female (58.6%) (Table 1). The majority of participants self-identified as white (62.9%) and more than half (54.3%) identified as Hispanic. Sixty percent of participants had a normal adult body mass index (BMI), while about a third of participants were either overweight (25.7%) or obese (10.0%). Most participants reported never smoking (64.3%), while 14.3% reported smoking in the past year. The majority of participants (61.4%) reported having a family history of any cardiovascular health issues, such as high blood pressure, angina, heart disease or myocardial infarction. Overall, key characteristics of these 70 individuals, including community of origin, race/ethnicity and sex, are similar to the distributions observed in the original subset of 737 individuals with carotid ultrasound in childhood (Table S1). Ambient and traffic-related air pollutant exposures varied across participants (Table S2) and correlations between all ambient and traffic-related pollutant measurements were also examined (Table S3).

**Table 1:** Selected demographic characteristics of CHS participants (*N* = 70)

	Mean (SD) or N (%)
Age at adult carotid ultrasound, years	21.9 (0.4)
Age at child carotid ultrasound, years	10.7 (0.4)
Change in age (time between ultrasounds), years	11.1 (0.3)
Sex	
Male	29 (41.4)
Female	41 (58.6)
Race	
White	44 (62.8)
More than one race	14 (20.0)
Other^a^	5 (7.2)
Unknown/not reported	7 (10.0)
Ethnicity	
Hispanic	38 (54.3)
Non-Hispanic	30 (42.9)
Unknown/not reported	2 (2.8)
CHS recruitment community	
Anaheim	7 (10.0)
Glendora	17 (24.3)
Long Beach	1 (1.4)
Mira Loma	5 (7.1)
Riverside	7 (10.0)
San Dimas	10 (14.3)
Santa Barbara	10 (14.3)
Upland	13 (18.6)
Parental Educational Attainment^b^	
High school or less	18 (25.7)
Some college or technical school	21 (30.0)
Completed college or more	31 (44.3)
Adult BMI (kg/m^2^)	24.8 (5.7)
BMI category	
Underweight (< 18.5)	3 (4.3)
Normal (18.5 to < 25)	42 (60.0)
Overweight (25 to < 30)	18 (25.7)
Obese (≥30)	7 (10.0)
Smoking status at young adult follow-up	
Never smoked	45 (64.3)
Smoked but not in past 12 months	15 (21.4)
Smoked in past 12 months	10 (14.3)
Any family history of CV events^c^	
No	27 (38.6)
Yes	43 (61.4)

^a^Other includes Asian, Pacific Islander, Alaska Native and American Indian

^b^ Parental education assessed prior to first CIMT measurement in childhood

^c^Events include hyperlipidemia (high cholesterol), hypertension (high blood pressure), stroke, coronary artery disease, and/or any other heart disease for any biological family members

To ensure comparability of measurements between outcome measurements performed across the ultrasound imaging systems, intra-class correlations were calculated for measures that were performed in duplicate for 29 individuals on both the GE and Siemens systems. Mean CIMT values for each ultrasound imaging system were very highly correlated (ICC = 0.993, range 0.985–0.996), indicating the comparability across machines and our ability to reliably compare CIMT from previously obtained images to current images (Table S4 and Figure S1). Reliability of measurements between image analysts was also calculated for a subset of images. The ICC for mean CIMT between image processors was very high (ICC = 0.9998), indicating that our image acquisition, processing and measurement methods produce highly reproducible and comparable estimates including between ultrasound imaging systems.

**Fig. 1:**
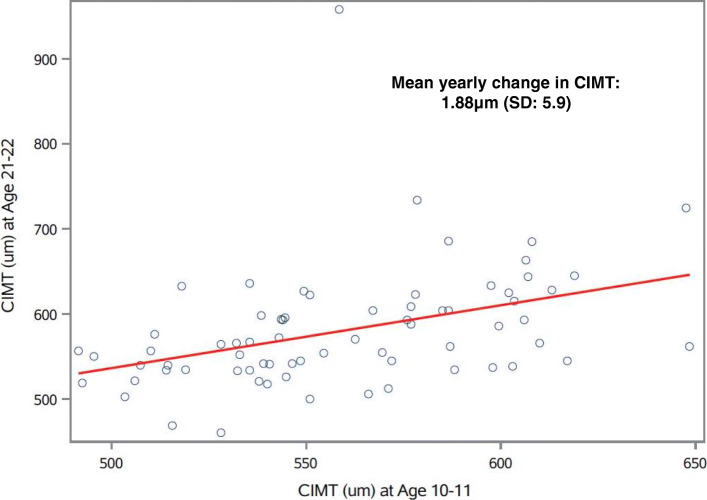
Correlation plot of CIMT ultrasound measures over time (*N* = 70). Open circles represent CIMT (μm) measurements obtained at age 10–11 years (x-axis) plotted against CIMT (μm) measurements obtained from the same participant at age 21–22 years of age

CIMT increased by 20.9 μm (SD: 65.9) (Table S5) on average between the age 10–11 years and 21–22 years measurements (Fig. 1), with an average yearly change in CIMT of 1.88 μm (SD: 5.9) over the follow-up period. Mean attained CIMT at age 21–22 among our 70 participants was similar (580.2 μm, SD: 70.9) (Table S5) to that of a Los Angeles university student population (603.4 μm, SD: 54.5) and within range of normal values previously observed for this age group [72].

We also explored yearly rate of CIMT change over the follow-up period by various participant characteristics. We did not observe statistically significant differences in CIMT change by sex, race, ethnicity, smoking or blood pressure (Table 2). However, we observed differences in CIMT among BMI categories, such that greater yearly increases in CIMT were observed among individuals who were obese in childhood and/or obese in adulthood, as compared to other BMI categories (Table 2). We also observed that individuals with a family history of CVD appeared to have a greater yearly increase in CIMT (2.52 μm/yr, SD: 6.79, *N* = 43) versus no history (0.85 μm/yr, SD: 3.87, *N* = 27) over the follow-up period, but these differences were not statistically significant.

**Table 2:** Descriptive statistics of overall yearly rate of change in CIMT (μm/year) from childhood (age 10–11 years) to adulthood (age 21–22 years) by participant characteristics (*N* = 70)

	N (%)	Mean Rate of Change CIMT (μm/year)	SD	*p*-value^a^
Sex				0.62
Male	29 (41.4)	1.45	4.92	
Female	41 (58.6)	2.18	6.50	
Race				0.60
White	44 (62.9)	1.73	3.48	
More than one race	14 (20.0)	1.30	4.05	
Other^b^	5 (7.1)	5.31	18.35	
Unknown/not reported	7 (10.0)	1.50	6.17	
Ethnicity				0.26
Hispanic	38 (54.3)	1.04	4.29	
Non-Hispanic	30 (42.8)	3.14	7.24	
Unknown/not reported	2 (2.9)	−1.14	9.15	
Parental Educational Attainment				0.34
High school or less	18 (25.7)	0.32	5.24	
Some college or technical school	21 (30.0)	1.72	3.83	
Completed college or more	31 (44.3)	2.89	7.16	
BMI category - child measurement				**0.01**
Underweight (< 18.5)	5 (7.1)	1.25	5.42	
Normal (18.5 to < 25)	44 (62.9)	1.51	4.02	
Overweight (25 to < 30)	14 (20.0)	0.02	3.55	
Obese (≥30)	7 (10.0)	8.35	13.21	
BMI category - adult measurement				**0.01**
Underweight (< 18.5)	3 (4.3)	2.74	1.32	
Normal (18.5 to < 25)	42 (60.0)	1.05	4.29	
Overweight (25 to < 30)	18 (25.7)	1.08	3.95	
Obese (≥30)	7 (10.0)	8.50	12.91	
Change in BMI category				0.27
Underweight/normal - no change	40 (57.1)	1.47	4.16	
Overweight/obese - no change	16 (22.9)	4.09	9.54	
Underweight/normal to overweight/obese	9 (12.9)	1.51	4.16	
Overweight/obese to underweight/normal	5 (7.1)	−1.33	3.79	
Change Direction in BMI Category				0.37
Decrease	10 (14.2)	−0.10	3.70	
No change	44 (62.9)	2.59	6.68	
Increase	16 (22.9)	1.15	4.20	
Child Systolic Blood Pressure				0.14
Below 75th percentile (< 106 mmHg)	52 (74.3)	1.27	3.91	
Above 75th percentile (≥106 mmHg)	18 (25.7)	3.62	9.46	
Adult Systolic Blood Pressure				0.59
Below 75th percentile (< 111 mmHg)	51 (67.9)	1.65	6.20	
Above 75th percentile (≥111 mmHg)	19 (27.1)	2.50	4.95	
Change in Systolic Blood Pressure				0.42
Below 75th percentile (< 8.7 mmHg change)	42 (60.0)	1.41	6.62	
Above 75th percentile (≥8.7 mmHg change)	28 (40.0)	2.57	4.53	
Smoking status				0.14
Never smoked	45 (64.3)	2.88	6.61	
Smoked but not in past 12 months	15 (21.4)	0.59	4.12	
Smoked in past 12 months	10 (14.3)	−0.69	2.96	
Any family history of CV events^c^				0.19
No	27 (38.6)	0.85	3.87	
Yes	43 (61.4)	2.52	6.79	

^a^As determined by t-test or one-way ANOVA; bold values represent *p* < 0.05

^b^Other includes Asian, Pacific Islander, Alaska Native and American Indian

^c^Events include hyperlipidemia (high cholesterol), hypertension (high blood pressure), stroke, coronary artery disease, and/or any other heart disease for any biological family members

We observed larger increases over time in CIMT with increased childhood exposure to traffic.

For example, greater yearly changes in CIMT between age 10–11 and age 21–22 were associated with exposure to traffic-related total NO_x_, such that a 1 SD increase in childhood total NO_x_ was associated with an average yearly increase of 2.17 μm (95% CI: 0.78, 3.56, *p* = 0.003) in CIMT over the follow-up period (Table 3 and Figure S2). Similarly, a 1 SD increase in childhood traffic-related freeway NO_x_ exposure was associated with an average yearly increase of 2.24 μm (95% CI: 0.84, 3.63, *p* = 0.002) in CIMT over the follow-up period (Table 3). Traffic density was related to statistically significant increases in CIMT over the follow-up, such that a 1 SD increase in traffic density was associated with a mean yearly increase of 2.11 μm (95% CI: 0.79, 3.43, *p* = 0.002) in CIMT. Childhood exposures to ambient pollutants (PM_10_, PM_2.5_, O_3_, and NO_2_) and traffic-related non-freeway NOx were not related to changes in CIMT. Further adjustment for additional potential confounders did not substantially change estimates or overall trends (Table S6).

**Table 3:** Estimates of association between ambient and traffic-related air pollutants and rate of change in CIMT (μm) per year from childhood to adulthood, adjusted for personal smoking, adult BMI and parental education (*N* = 70)

	Estimate (95% CI) per 1 SD increase in exposure^**a**^	***P***-value
**Ambient Pollutants (monthly average)**		
Daily 8-h max O_3_ concentration (ppb)	−1.02 (− 2.41, 0.36)	0.14
24-h average PM_2.5_ concentration (μg/m^3^)	− 0.40 (− 1.75, 0.96)	0.56
24-h average PM_10_ concentration (μg/m^3^)	−0.51 (− 1.85, 0.85)	0.46
24-h average NO_2_ concentration (μg/m^3^)	0.17 (−1.19, 1.53)	0.81
**Local Traffic-Related Pollutants/Measures**		
Total NOx (ppb)	2.17 (0.78, 3.56)	**0.003**
Freeway NOx (ppb)	2.24 (0.84, 3.63)	**0.002**
Non-Freeway NOx (ppb)	0.60 (−0.78, 1.98)	0.39
Traffic Density (within 300 m buffer)	2.11 (0.79, 3.43)	**0.002**

^a^Estimates are each scaled to represent yearly change in CIMT relative to a 1 standard deviation (SD) increase in childhood exposure to the corresponding pollutant

We also observed higher attained CIMT in early adulthood in relation to childhood exposure to traffic density and traffic-related measures of NOx (Table 4). Total NOx and freeway NOx both were positively associated with attained CIMT at age 21–22 years, such that 1 SD increase in childhood traffic-related total NOx was associated with was associated with 20.60 μm (95% CI: 2.87, 38.36, *p* = 0.02) in attained CIMT and a 1 SD increase in childhood traffic-related freeway NO_x_ exposure was associated with 21.09 μm (95% CI: 3.33, 38.85, *p* = 0.02) in attained CIMT. We observed a 23.01 μm (95% CI: 6.38, 39.42; *p* = 0.007) higher attained CIMT in early adulthood for each SD increase in traffic density. Ambient pollutants and non-freeway NOx were not related to attained CIMT in early adulthood.

**Table 4:** Estimates of association between ambient and traffic-related air pollutants and adult attained^a^ CIMT (μm), adjusted for personal smoking, adult BMI, and parental education (*N* = 70)

	Estimate (95% CI) per 1 SD increase in exposure^**b**^	***P***-value
**Ambient Pollutants (monthly average)**		
Daily 8-h max O_3_ concentration (ppb)	−10.35 (− 27.54, 6.83)	0.23
24-h average PM_2.5_ concentration (μg/m^3^)	−1.62 (− 18.38, 15.14)	0.85
24-h average PM_10_ concentration (μg/m^3^)	−2.81 (− 19.54, 13.90)	0.74
24-h average NO_2_ concentration (μg/m^3^)	6.43 (− 10.30, 23.16)	0.45
**Local Traffic-Related Pollutants/Measures**		
Total NOx (ppb)	20.60 (2.87, 38.36)	**0.02**
Freeway NOx (ppb)	21.09 (3.33, 38.85)	**0.02**
Non-Freeway NOx (ppb)	6.25 (−10.84, 23.33)	0.47
Traffic Density (within 300 m buffer)	23.01 (6.38, 39.42)	**0.007**

^a^Adult attained CIMT represents absolute value of CIMT measured at time of young adult assessment

^b^Estimates are each scaled to represent a change in beta for a 1 standard deviation (SD) increase in exposure to the corresponding pollutant

In exploratory analyses stratified by sex, we observed somewhat greater increases in CIMT over time in female participants versus male participants, in relation to traffic-related NOx (Table S7). Among female participants, a 1 SD increase in childhood total NO_x_ was associated with an average yearly increase of 3.07 μm (95% CI: 0.83, 5.38, *p* = 0.009) in CIMT over the follow-up period, as compared to males, for whom childhood total NO_x_ was associated with an average yearly increase of 1.12 μm (95% CI: − 0.65, 2.91, *p* = 0.20) in CIMT. Similarly, freeway NOx was associated with greater changes in CIMT among females as compared to males. However, traffic density was associated with statistically significant yearly increases in CIMT in both females 2.51 μm (95% CI: − 0.21, 4.81, *p* = 0.03) and in males 1.62 μm (95% CI: 0.06, 3.24, *p* = 0.04).

## Discussion

In this study of 70 young adults with repeated carotid artery ultrasound images from age 10–11 and age 21–22, we observed associations between traffic density and traffic-related NOx (total and freeway-related) with CIMT, a measure of subclinical atherosclerosis. Our results suggest that childhood exposure to local traffic-related air pollution is associated with greater rate of yearly change in CIMT from childhood to adulthood, as well as greater attained CIMT levels by age 21–22 with adjustment for adult BMI and personal smoking. Further, in exploratory analyses stratified by sex, somewhat greater yearly increases in CIMT were observed with total NOx and freeway NOx among female participants. However, sex-stratified analyses should be interpreted with caution given the small sample size in each group. Our results also suggest that individual factors, such as BMI, may influence early patterns of progression of subclinical atherosclerosis and cardiovascular health in early adulthood.

While overt CVD is rarely diagnosed until adulthood, early indicators of pathogenic processes can be observed in childhood [73]. Atherosclerosis is a lifelong process that begins in childhood and a number of studies, including many utilizing carotid artery ultrasound measures, have observed changes in childhood associated with later life cardiovascular health in adulthood [50, 74–82]. Air pollution is thought to promote CVD pathogenesis by affecting key cellular pathways involved in inflammation, vascular remodeling, lipid deposition, fibrosis and calcification, and studies among children and young adults have observed that air pollutant levels are related to greater inflammation, endothelial damage and oxidative stress [83–85]. In recent years, numerous epidemiological studies have provided strong evidence that exposure to air pollutants not only increases CVD incidence, but also accelerates atherosclerosis in adults [8, 9, 14–27, 32, 35]. Despite the considerable support linking air pollution and CVD in adults, very little is known about the origins of CVD in early stages during childhood and adolescence, particularly in relation to environmental exposures, such as air pollution. Even in this relatively small study population, we observed statistically significant changes in CIMT in relation to traffic-related exposure markers in healthy young adults and while these changes may appear small, small increases in CIMT over the course of the lifetime may lead to greater thickening over time and greater long-term risk of CVD.

We found that residential traffic density and exposure to traffic-related NOx in childhood were related to greater changes in CIMT over time. While we previously found no association between traffic-related air pollution in early childhood and CIMT at age 10 years [21], a limited number of other studies have shown relationships between traffic-related exposures and early cardiovascular changes in children and young adults [21, 36–39]. One study found increased carotid artery stiffness among 6–14 year old children living in close proximity (< 300 m) to a highly-trafficked roadway, as compared to those living more than 300 m from the road, while a similar study found that children residing less than 100 m from a busy road had 15% increased CIMT compared to those who lived further from traffic [37, 40]. Recent work found that exposures to NO_2_, NO_x_ and PM were associated with decreased distensibility in children at age 5 [39]. Others have suggested that prenatal and early life may represent sensitive periods for air pollution exposure and cardiovascular health, and have observed associations between prenatal and early air pollution exposures and adverse changes in childhood cardiovascular risk factors, including CIMT, carotid artery stiffness, BP and BMI [21, 22, 36, 41–46]. Although a number of these prior studies observed associations with ambient air pollutants, including O_3_ and PM_2.5_, we did not observe any relationship between ambient pollutants and CIMT in our analysis [21, 22, 36, 42–44]. Instead, we found consistent associations with measures of more spatially variable and local exposure to traffic-related air pollution (CALINE4 NOx overall and from freeways and traffic density). Ambient air pollutants, assigned by interpolating from regulatory monitoring network data capture the regional, background levels of air pollution over the study area. Several sources contribute to these levels, and these sources could be emitted or formed locally or transported into a city’s airshed from upwind sources for example (including traffic in the region). However, the ambient measures are not attributed to any specific source(s). They represent overall levels and are mainly capturing temporal variability in ambient air pollution (from all sources in an airshed). Whereas the traffic-related NOx estimates are calculated using a deterministic line source dispersion model (CALINE4), which captures the high spatial variability in traffic emissions from nearby roadways and their dispersion patterns.

Therefore, the traffic metrics model the contribution of traffic sources specifically (by road class) to NOx levels at residences and could be capturing the effects of the near-roadway air pollution mixture that is enriched in ultrafine and coarse particles, elemental and organic carbon, metals, nitrogen oxides and other tailpipe and non-tailpipe components of traffic emissions that have been shown to be associated with inflammation, oxidative stress and cardiovascular endpoints including CIMT [86–88]. In an analysis of PM samples collected within eight of the CHS communities, CALINE4 freeway NOx was a strong predictor of tailpipe (diesel) and non-tailpipe (abrasive vehicular emissions) particulate matter source contributions in the PM_0.2_ (quasi-ultrafine), PM_2.5_ (fine) and PM_2.5–10_ (coarse) size fractions [89]. Overall, our findings suggest the need to investigate characteristics of the near-roadway traffic mixture (e.g., chemical composition, size distribution, solubility, etc.) that could be driving these subclinical effects [90].

Importantly, childhood cardiovascular risk factors, including carotid artery stiffness, blood lipids, BP and BMI, have been associated with increases in CIMT in later life [47–50]. However, no studies have characterized changes in measures of subclinical atherosclerosis over time in younger populations, or the role of air pollutants in these changes. To put our results in context, we observed a change of ~ 1.7 μm/year change in CIMT associated with a 1 SD increase in residential traffic density, which is very similar to previously observed change in CIMT of 3 μm over a ~ 2 year period related to a 10 mmHg increase in SBP in 14–18 year olds [91]. The rate of change we observed with traffic-related exposure also was similar to previously observed associations with age in a somewhat older cohort of young adults over six years of follow up in the Young Finns study (ages 24–39 at baseline) [92]. Age was associated with a total CIMT change of 14.5 μm over follow up, equivalent to approximately a 2.4 μm/year change in CIMT [92]. To our knowledge, ours is the first study to examine changes over time in CIMT in relation to air pollution during the critical transition period from childhood into adulthood when many lifelong health patterns and habits become established.

In our study, we observed that higher BMI in both childhood and adulthood were related to a significant mean increase in CIMT over time in univariate analyses. Other studies have observed relationships between cardiovascular risk factors, including BMI, and atherosclerosis-related vascular remodeling and arterial stiffness in adults and increasingly, the presence of these risk factors in childhood has been linked to measurements of atherosclerosis in adulthood [3, 47, 48, 74, 93–95]. A combined study of 4 international cohorts found that BMI, BP, smoking and blood lipids were independent predictors of CIMT in early adulthood [49]. Some risk factors have also been associated with CIMT in children, including BP, family history of hypertension, and markers of insulin resistance and exposure to tobacco smoke, suggesting that cardiovascular stressors may lead to early subclinical atherosclerosis [81, 82, 96–101]. While BP and family history of CVD were not associated with statistically significant changes in CIMT in the current study, we will continue to explore these risk factors in future work. Overall, little is known about how individual factors may influence changes in CIMT in the transition from childhood to adulthood and further investigation is warranted.

Our study has a number of strengths. We were able to examine changes in measures of subclinical atherosclerosis over time in a unique dataset from the CHS that captures residential mobility over childhood and includes detailed ambient and traffic-related air pollution exposure estimates, in addition to repeated carotid artery ultrasound measures in childhood and in early adulthood. One key advantage of this study is that the same ultrasound imaging technician who performed the initial carotid artery ultrasound imaging of CHS participants at age 10–11 conducted all repeat measures for this project, eliminating inter-technician differences in image acquisition and providing consistency across our time points, despite the intervening years. Further, the imaging technician had access to prior images to measure within the same anatomical landmarks. Imaging technicians and processors were blinded to all exposure information to avoid biasing outcome measurements. Our study also has some limitations. First, our sample size in this study was limited and we were not well powered to investigate effect modification by potentially important factors, such as BMI and sex. Despite the small sample size, we observed consistent, statistically significant associations between traffic-related exposures and CIMT and suggestive differences by sex in stratified analyses. However, these results must be interpreted with caution given the small samples size and future studies with a larger sample will be able to more fully evaluate the possible impact of other factors on change in CIMT. We also cannot rule out unmeasured confounding and it is possible that other factors, such as dietary intake and physical activity may impact patterns of subclinical atherosclerosis progression over time and should be taken into account in future studies. Also, we adjusted for socioeconomic status (SES) using parental education as a proxy variable, which may not fully capture SES variability or changes since time of assessment of this variable in childhood. Given the age of these participants, many are still continuing their education (either full or part-time) and may live with parents or roommates, making assessment of SES of the participant by more traditional indicators, such as educational attainment or household income, unreliable measures of SES. While this pilot study was conducted among a convenience sample of 70 CHS participants, the distribution of original enrollment communities and demographic characteristics of these participants were similar to the overall distributions of the original cohort of CIMT participants. Further, we did not have complete modeled air pollution data beyond age 10 years for all participants at the time of this analysis to account for lifetime exposures. However, exploratory analyses that included air pollution exposures up to age 16 among those with these exposure data (not shown) did not substantially alter our findings and future work will further explore lifetime air pollutant exposures.

## Conclusions

In conclusion, our study results suggest that traffic-related air pollutants may be associated with adverse changes in CIMT, a measure of subclinical atherosclerosis, over time in a population of young adults who have been followed since childhood. Such increases in progression of CIMT by early adulthood could indicate greater CIMT thickening over time and may be relevant in predicting long-term CVD risk. While our results must be interpreted with caution given the limited sample size, the statistically significant associations between traffic-related air pollutants and change in CIMT suggest important changes during this critical lifestage that warrant further studies with larger study samples. Early detection of initial vascular changes in childhood into young adulthood with carotid artery ultrasound measurements has the potential to not only improve our understanding of CVD pathogenesis as it relates to air pollution exposures, but also facilitates identification of children at risk for CVD later in life*.*

## Supplementary Information

**Additional file 1.**

## Data Availability

The datasets used and analyzed during the current study are available from the corresponding author on reasonable request.

## Abbreviations

CVD: Cardiovascular disease
CIMT: Carotid intima media thickness
CHS: Children’s Health Study
O_3_: Ozone
NO_2_: Nitrogen dioxide
NO_x_: Nitrogen oxides
PM: Particulate matter
