# Correction: Childhood traffic-related air pollution and adverse changes in subclinical atherosclerosis measures from childhood to adulthood

**DOI:** 10.1186/s12940-022-00931-2

**Published:** 2022-11-16

**Authors:** Shohreh F. Farzan, Rima Habre, Phoebe Danza, Frederick Lurmann, W. James Gauderman, Edward Avol, Theresa Bastain, Howard N. Hodis, Carrie Breton

**Affiliations:** 1 Department of Preventive Medicine, Keck School of Medicine of University of Southern California, 2001 N. Soto Street, Los Angeles, CA 90089 USA; 2 Sonoma Technology Inc, Petaluma, CA USA; 3 Department of Medicine, Keck School of Medicine of University of Southern California, Los Angeles, CA 90089 USA; 4 Atherosclerosis Research Unit, University of Southern California, Los Angeles, CA 90089 USA

**Correction: Environ Health 20, 44 (2021)**

**https://doi.org/10.1186/s12940-021-00726-x**

Following the publication of the original article [1], the author reported that they listed the incorrect machine model for the images that were obtained. The published version of the methods states:

In brief, high resolution B-mode ultrasound carotid artery images were acquired using a Siemens Acuson ***CV70*** (Mountain View, CA) ultrasound imaging system using a linear array ***7.5 MHz*** transducer .

However, the correct information should state:

In brief, high resolution B-mode ultrasound carotid artery images were acquired using a Siemens Acuson ***X 300*** (Mountain View, CA) ultrasound imaging system using a *linear array****VF 10-5 (8.0 MHz)*** transducer .
