# Supplementary material for: Childhood traffic-related air pollution and adverse changes in subclinical atherosclerosis measures from childhood to adulthood
Source: Environ Health. 2021 Apr 14;20:44. doi: 10.1186/s12940-021-00726-x (PMC8048028; doi:10.1186/s12940-021-00726-x)
Supplement: Supplementary file 1 — Additional file 1. [file 12940_2021_726_MOESM1_ESM.docx]

**Farzan et al.**

**Supplemental Tables**

| **Table S1. Comparison of selected demographic characteristics of CHS participants in current study (N=70) versus overall CIMT study sample (N=737)** | | |  |
| --- | --- | --- | --- |
|  | Current study  (N=70)  N (%) | Overall CIMT study (N=737)  N (%) |  |
| Age at child carotid ultrasound, years | 10.7 (0.4) | 11.3 (0.6) |  |
| Sex |  |  |  |
| Male | 29 (41.4) | 358 (49.0) |  |
| Female | 41 (58.6) | 379 (51.0) |  |
| Race |  |  |  |
| White | 44 (62.8) | 313 (42.5) |  |
| More than one race | 14 (20.0) | 175 (23.7) |  |
| Other/Unknown^a^ | 13 (17.2) | 249 (33.8) |  |
| Ethnicity |  |  |  |
| Hispanic | 38 (54.3) | 413 (56.0) |  |
| Non-Hispanic | 30 (42.9) | 305 (41.4) |  |
| Unknown/not reported | 2 (2.8) | 19 (2.6) |  |
| CHS recruitment community |  |  |  |
| Anaheim | 7 (10.0) | 78 (10.6) |  |
| Glendora | 17 (24.3) | 119 (16.2) |  |
| Long Beach | 1 (1.4) | 36 (4.9) |  |
| Mira Loma | 5 (7.1) | 97 (13.2) |  |
| Riverside | 7 (10.0) | 82 (11.1) |  |
| San Dimas | 10 (14.3) | 101 (13.7) |  |
| Santa Barbara | 10 (14.3) | 115 (15.6) |  |
| Upland | 13 (18.6) | 109 (14.8) |  |
| ^a^Other includes Asian, Black, Pacific Islander, Alaska Native and American Indian | | |  |

| **Table S2. Ambient and traffic-related air pollutant exposures among CHS participants from birth to time of childhood ultrasound assessment (N=70)** | | |
| --- | --- | --- |
|  | Mean (SD) | Range |
| **Ambient Pollutants (monthly average)** |  |  |
| Daily 8-hour max O_3_ concentration (ppb) | 42.3 (4.3) | 30.3-52.9 |
| 24-hour average PM_2.5_ concentration (µg/m^3^) | 19.2 (4.2) | 8.7-25.4 |
| 24-hour average PM_10_ concentration (µg/m^3^) | 43.3 (10.3) | 20.6-63.7 |
| 24-hour average NO_2_ concentration (µg/m^3^) | 25.7 (6.5) | 10.6- 34.5 |
| **Local Traffic-Related Pollutants/ Measures** |  |  |
| Total NOx (ppb) | 24.7 (22.4) | 1.5-117.4 |
| Freeway NOx (ppb) | 18.9 (20.9) | 0.001-108.0 |
| Non-Freeway NOx (ppb) | 5.9 (3.2) | 1.4-18.9 |
| Traffic Density (within 300m buffer) | 77.2 (104.6) | 1.4-605.7 |

| **Table S3. Spearman correlations for ambient and traffic-related air pollutant exposure measurements (N=70).** | | | | | | | | |
| --- | --- | --- | --- | --- | --- | --- | --- | --- |
|  | Daily 8-hr max O_3_ (ppb) | 24-hr average PM_2.5_ (µg/m^3^) | 24-hr average PM_10_ (µg/m^3^) | 24-hr average NO_2_ (µg/m^3^) | Total NOx (ppb) | Freeway NOx (ppb) | Non-Freeway NOx (ppb) | Traffic Density (within 300m) |
| Daily 8-hr max O_3_ (ppb) | 1 |  |  |  |  |  |  |  |
| 24-hr average PM_2.5_ (µg/m^3^) | 0.71*** | 1 |  |  |  |  |  |  |
| 24-hr average PM_10_ (µg/m^3^) | 0.64*** | 0.97*** | 1 |  |  |  |  |  |
| 24-hr average NO_2_ (µg/m^3^) | -0.003 | 0.39** | 0.45*** | 1 |  |  |  |  |
| Total NOx (ppb) | -0.22 | -0.19 | -0.18 | -0.09 | 1 |  |  |  |
| Freeway NOx (ppb) | -0.22 | -0.10 | -0.06 | -0.02 | 0.96*** | 1 |  |  |
| Non-Freeway NOx (ppb) | -0.28* | -0.47*** | -0.52*** | -0.21 | 0.59*** | 0.41** | 1 |  |
| Traffic Density (within 300m) | -0.35** | -0.44** | -0.42** | 0.02 | 0.76*** | 0.67*** | 0.64*** | 1 |
| *p<0.05  ** p<0.01  ** p<0.0001 | | | | | | | | |

| **Table S4. Correlation between CIMT Ultrasound Measurements Between GE and Siemens Machines (N=29)** | | | | |
| --- | --- | --- | --- | --- |
| CIMT Values | N | ICC | Lower Bound | Upper Bound |
| All (2 measurements per subject, per machine)^a^ | 29 participants,  116 images | 0.990 | 0.983 | 0.994 |
| Mean of 2 CIMT measurements, per machine^a^ | 29 participants, 58 images | 0.993 | 0.985 | 0.996 |
| ^a^ Each participant received two measurements of CIMT with each ultrasound machine for a total of four measurements. ICCs were calculated for both unaveraged values (all) or for averaged values from each instrument (mean).  ^b^ Two analysts were given duplicate mages from 5 participants (10 images each) and asked to provide measurements of each. ICC was calculated between all measurements from each analyst. | | | | |

| **Table S5. Carotid artery ultrasound measurements at child visit, adult visit, and change from child to adult (N=70).** | | | | |
| --- | --- | --- | --- | --- |
|  | **Mean** | **SD** | **Median** | **IQR** |
| **Child measurements, age 10-11 years** |  |  |  |  |
| Intima-media thickness, right side (µm) | 559.25 | 37.81 | 551.00 | 54.00 |
| Systolic blood pressure, avg (mmHg) | 99.51 | 7.37 | 100.17 | 13.67 |
| Diastolic blood pressure, avg (mmHg) | 55.30 | 5.78 | 55.33 | 8.67 |
| **Adult measurements, age 21-22 years** |  |  |  |  |
| Intima-media thickness, right side (µm) | 580.16 | 70.88 | 565.00 | 70.00 |
| Systolic blood pressure, avg (mmHg) | 106.06 | 7.56 | 105.00 | 10.00 |
| Diastolic blood pressure, avg (mmHg) | 59.39 | 4.22 | 58.00 | 5.00 |
| **Change from child to adult measurements** |  |  |  |  |
| Change in intima-media thickness (µm) | 20.91 | 65.88 | 18.25 | 59.50 |
| Change in Systolic blood pressure, avg (mmHg) | 6.55 | 8.89 | 7.00 | 12.67 |
| Change in Diastolic blood pressure, avg (mmHg) | 4.08 | 5.95 | 4.00 | 9.00 |

| **Table S6. Estimates of association between ambient and traffic-related air pollutants and rate of change in CIMT (µm) per year from childhood to adulthood, adjusted for personal smoking, adult BMI, parental education, sex, ethnicity, family history of CVD and systolic blood pressure change over time (N=70).** | | | | | |
| --- | --- | --- | --- | --- | --- |
|  | | **Estimate (95% CI)**  **per 1 SD increase in exposure^a^** | | **P-value** | |
| **Ambient Pollutants (monthly average)** | | | | | |
| Daily 8-hour max O_3_ concentration (ppb) | | | -1.10 (-2.54, 0.33) | | 0.13 |
| 24-hour average PM_2.5_ concentration (µg/m^3^) | | | -0.67 (-2.15, 0.83) | | 0.38 |
| 24-hour average PM_10_ concentration (µg/m^3^) | | | -0.72 (-2.17, 0.72) | | 0.33 |
| 24-hour average NO_2_ concentration (µg/m^3^) | | | -0.03 (-1.55, 1.49) | | 0.97 |
| **Local Traffic-Related Pollutants/Measures** | | | | | |
| Total NOx (ppb) | 2.13 (0.67, 3.58) | | | | 0.005 |
| Freeway NOx (ppb) | 2.18 (0.72, 3.64) | | | | **0.004** |
| Non-Freeway NOx (ppb) | 0.64 (-0.78, 2.04) | | | | 0.37 |
| Traffic Density (within 300m buffer) | 2.20 (0.73, 3.56) | | | | **0.003** |
| ^a^Estimates are each scaled to represent yearly change in CIMT relative to a 1 standard deviation (SD) increase in childhood exposure | | | | | |

| **Table S7. Sex-stratified estimates of association between ambient and traffic-related air pollutants and rate of change in CIMT (µm) per year from childhood to adulthood, adjusted for personal smoking, parental education, and adult BMI.** | | | | |
| --- | --- | --- | --- | --- |
|  | | | | |
|  | **Female Participants**  **N=41** | | **Male Participants**  **N=29** | |
|  | **Estimate (95% CI) per 1 SD increase in exposure^a^** | **P-value** | **Estimate (95% CI)**  **per 1 SD increase in exposure^a^** | **P-value** |
| **Ambient Pollutants (monthly average)** | | | | |
| Daily 8-hour max O_3_ concentration (ppb) | -0.56 (-2.59, 1.51) | 0.59 | -1.16 (-3.23, 0.95) | 0.27 |
| 24-hour average PM_2.5_ concentration (µg/m^3^) | -0.34 (-2.48, 1.77) | 0.75 | 0.14 (-1.77, 2.02) | 0.88 |
| 24-hour average PM_10_ concentration (µg/m^3^) | -0.62 (-2.58, 1.34) | 0.53 | 0.31 (-1.80, 2.47) | 0.76 |
| 24-hour average NO_2_ concentration (µg/m^3^) | 0.02 (-2.09, 2.15) | 0.98 | 0.57 (-1.18, 2.35) | 0.51 |
| **Local Traffic-Related Pollutants/Measures** | | | | |
| Total NOx (ppb) | 3.07 (0.83, 5.38) | **0.009** | 1.12 (-0.65, 2.91) | 0.20 |
| Freeway NOx (ppb) | 3.35 (0.92, 5.59) | **0.007** | 1.19 (-0.56, 2.93) | 0.18 |
| Non-Freeway NOx (ppb) | 0.94 (-0.84, 2.71) | 0.29 | -0.19 (-2.72, 2.33) | 0.87 |
| Traffic Density (within 300m buffer) | 2.51 (0.21, 4.81) | **0.03** | 1.62 (0.06, 3.24) | **0.04** |
| ^a^Estimates are each scaled to represent yearly change in CIMT relative to a 1 standard deviation (SD) increase in childhood exposure | | | | |


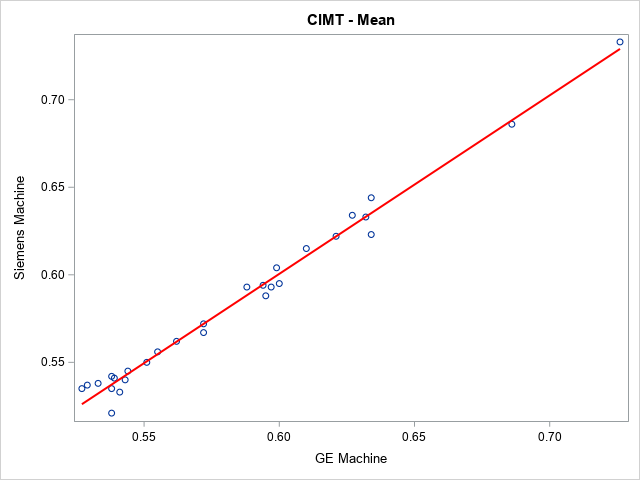


**CIMT (μm) measured with GE LOGIQ**

**CIMT (μm) measured with Siemens Acuson**

ICC: 0.993 (0.985-0.996)

**Figure S1**. Comparability of GE LOGIQ and Siemens Acuson CV70 ultrasound imaging systems. Duplicate ultrasound images were obtained from N= 29 young adult participants at the in-person visit using both the GE LOGIQ and Siemens Acuson CV70 ultrasound imagers. CIMT measurements were obtained in duplicate on each machine (4 total measures per subject). Average CIMT values for are plotted for each machine.

**Estimated Yearly Change in CIMT (μm)**

**Total NOx (ppb) from local traffic**


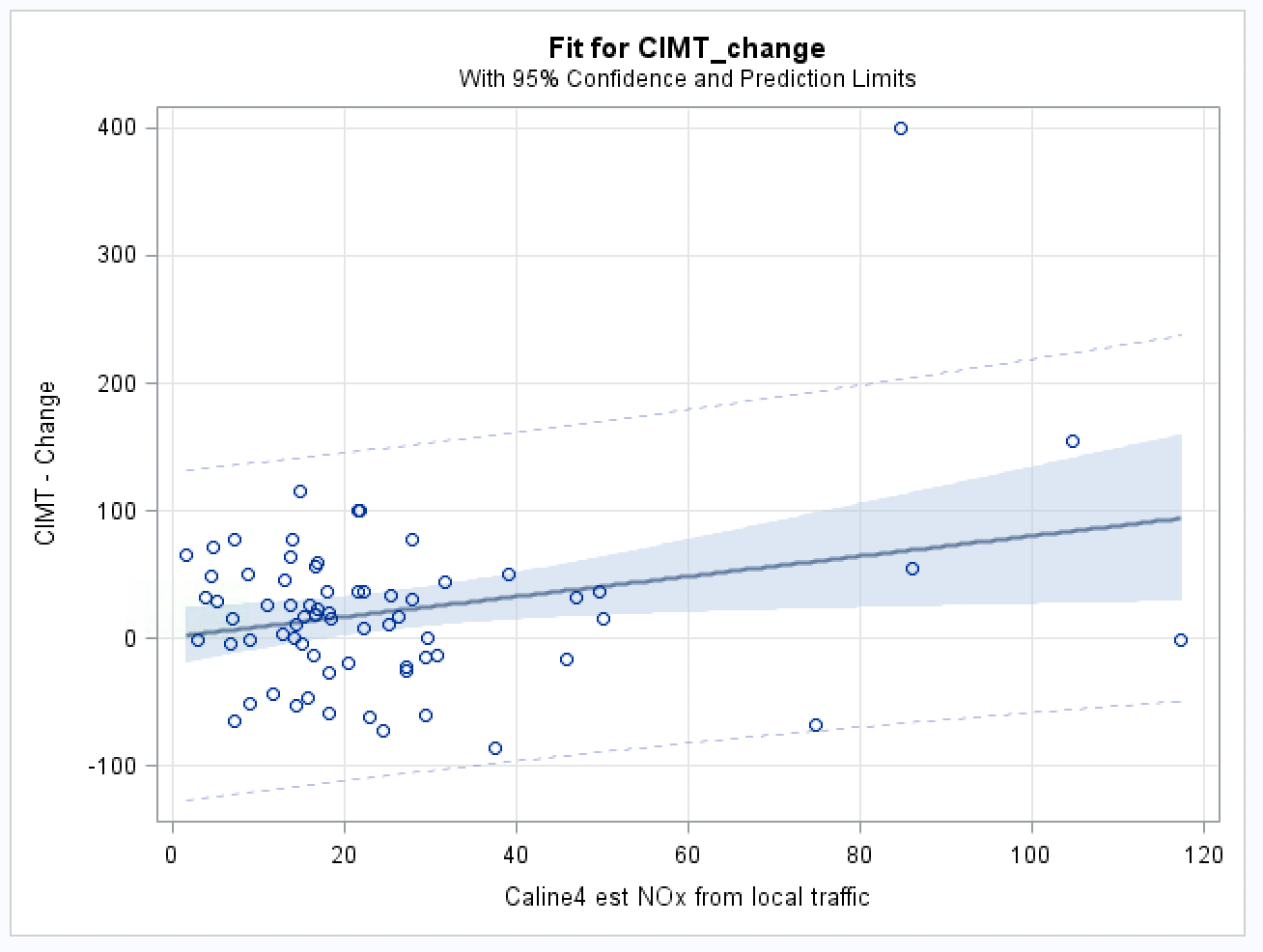


**Figure S2**. Univariate association of total NOx (ppb) from local traffic with yearly change in CIMT measurements. Blue shading represents 95% confidence interval.
